# Diagnostic Accuracy of Subjective Features and Physical Examination Tests for Morton Neuroma: A Systematic Review

**DOI:** 10.1177/24730114241291055

**Published:** 2024-11-19

**Authors:** Mark Pitcher, Andrea Moulson, David Pitcher, Anthony Herbland, Grad Cert, Mindy C. Cairns

**Affiliations:** 1 Department of Allied Health Professions, Midwifery and Social Work, University of Hertfordshire, Hatfield, United Kingdom; 2 The Renal Association, Filton, Bristol, United Kingdom

**Keywords:** Morton neuroma, Diagnosis, Sensitivity, Specificity, Systematic review

## Abstract

**Background:**

Morton neuroma (MN) is a common pathology with many reported subjective features and physical examination tests. The objective of this systematic review was to examine the diagnostic accuracy of subjective features and physical examination tests for MN.

**Methods:**

CINAHL, CENTRAL, EMBASE, MEDLINE, PubMed, Opengrey, PEDro, PsycINFO, Scopus and Trials register were searched in January 2021. Two reviewers independently screened studies for inclusion using the following criteria: (1) prospective or retrospective cohort studies, (2) participants aged ≥18 years with suspected MN, (3) primary data allowing construction of 2 × 2 diagnostic table or reported sensitivity and specificity figures, (4) diagnosis of MN using magnetic resonance imaging, ultrasonography, surgery, positive response to steroid and/or anesthetic injection, and (5) in English or translatable. Quality of included studies was assessed using the Quality Assessment for Diagnostic Accuracy Studies version-2 (QUADAS-2) tool.

**Results:**

The search yielded 1170 results, of which 9 were included in this systematic review. Narrative synthesis revealed that subjective clicking reported by a patient was highly specific (0.96) and had a high positive likelihood ratio (13.14). The modified webspace tenderness test (thumb index finger squeeze test) was highly sensitive (0.96) with a low negative likelihood ratio (0.04). The commonly reported feeling of “walking on a pebble” and “burning pain” had sensitivities of 43% to 53% and 54% to 57% and associated specificities of 52% and 48%, respectively.

Only 1 study had low risk of bias. The review was limited by the number of studies that included few or no patients without MN, and the impact this had on the ability to calculate diagnostic accuracy.

**Conclusion:**

There is strong evidence that clicking reported by a patient rules in MN and that the modified webspace tenderness test rules out MN when negative.

## Background

Morton neuroma (MN) is a painful forefoot condition involving entrapment of the common plantar digital nerve in the intermetatarsal space.^23^ MN is not a true neuroma^5^ as is it degenerative rather than neoplastic,^31^ with histologic findings including neural degeneration, perineural fibrosis, arterial degeneration, and edema of the endoneurium.^16,19,30^ MNs have been reported in all 4 intermetatarsal spaces, with the third space most commonly affected (reported range 43%-86.4%), followed by the second (range 5.1%-57%).^44,47^ MN is more common in females (87.5 cases per 100 000 of UK population) compared with males (50.2 per 100 000)^24^ and is the third most common condition referred to foot and ankle surgeons in the United Kingdom.^20^

Diagnosis of MN is predominantly based on clinical assessment findings, including typical subjective features and physical examination tests, with other modalities (ultrasonography, magnetic resonance imaging [MRI], response to anesthetic/steroid injection) used to confirm diagnosis or exclude other pathologies.^5,15,18^ Both ultrasonography and MRI have been shown to have high levels of sensitivity and specificity in the diagnosis of MN.^7^ Some authors have argued that routine imaging for MN is a waste of resources^41^ and that imaging should only be used where clinical findings are inconclusive.^36^ More ultrasonography scans are ordered for MN than any other foot and ankle pathology in the United Kingdom,^20^ and the combined cost of MRI and ultrasonography scans for 179 suspected MNs in the USA was estimated to be $134 900 (£98 548).^38^ NICE^32^ clinical knowledge summaries on diagnosis of MN advise that further investigations are generally not necessary, indicating that initial clinical diagnosis is very important. Clinical diagnosis of MN, however, can be difficult,^22^ with reported accuracy rates varying from 58% to 93%.^6,41^

Symptoms commonly reported as being associated with MN include burning pain in the forefoot that can become debilitating and limit walking, altered sensation or numbness in the distribution of the affected nerve, and a feeling of walking on a pebble.^5,34,46^ Commonly recommended tests include Mulder sign, webspace tenderness, and foot squeeze tests.^13^ Although subjective features and physical examination tests for MN are discussed in review articles^5,18^ and recommended by a 4-round Delphi consensus study completed by 16 expert health professionals,^13^ there are no known systematic reviews of their diagnostic accuracy. The aim of this systematic review was to determine the diagnostic accuracy of subjective features and physical examination tests for MN that may facilitate accurate and timely diagnosis.

## Methods

### Registration and Searches

This study was registered with the International Prospective Register of Systematic Reviews (PROSPERO). The study was completed using the Preferred Reporting Items for Systematic Reviews and Meta-Analyses (DTA PRISMA) guidelines that have been adapted for systematic reviews of diagnostic test accuracy.^28^

CINAHL, CENTRAL, EMBASE, MEDLINE, PubMed, Opengrey, PEDro, PsycINFO, Scopus, and Trials register were searched from inception to January 12, 2021. Search terms were derived by compiling a list of all possible synonyms for MN during the scoping review. To enhance face validity of terms, a group of 16 senior musculoskeletal physiotherapists along with an orthopaedic surgeon with an interest in foot and ankle disorders and research were sent the list to add appropriate terms. Search terms for index tests, reference standards, and accuracy were developed through reading systematic reviews that investigated diagnostic test accuracy in other conditions^1,39,40,43^ as well as from MN articles during the scoping review. Hand-searching of included studies was also undertaken as well as forward citation searching using Google Scholar.^8^ The search strategy can be found in Appendix A.

### Study Selection

Prospective and retrospective cohort studies were included if they met all of the following criteria: included patients aged ≥18 years with suspected MN; presented primary data on subjective features of MN and/or results of the outcome of a physical examination test in enough detail to allow construction of the 2 × 2 diagnostic table or reported sensitivity and specificity figures; diagnosis of MN was confirmed using either MRI, ultrasonography, surgery, positive response to steroid and/or anesthetic injection (either unguided or ultrasonography guided); and were in English or translatable into English. Systematic reviews, case studies, or series and cadaver studies were excluded.

Two reviewers (MP, DP) independently performed searches and independently screened titles, abstracts, and full texts for eligibility. Full texts were retrieved for studies that were deemed eligible or where insufficient information was included in the abstract to determine eligibility. Full texts were screened independently using the above criteria by the same 2 reviewers with further information or clarification requested from authors via email where required. A third reviewer was available to make a final decision in the event of disagreement; however, this was not required.

### Data Extraction and Quality Assessment

Data on study details, patient demographics, and results of index tests and reference standards were extracted independently by 2 reviewers (MP, DP) using a data extraction form based on Cochrane recommendations.^25^ Next, 2 × 2 diagnostic tables for each subjective feature and physical examination test in all studies were created from extracted data. Disagreements were resolved through consensus between the 2 reviewers, with a third reviewer available if necessary.

The QUADAS-2 risk of bias tool^48^ has been specifically designed for use in systematic reviews of quality of diagnostic accuracy studies and is recommended for such use by Cochrane.^49^ QUADAS-2 has been used in other systematic reviews of diagnostic test accuracy^9,40^ and was completed independently by 2 reviewers (MP, DP) to assess risk of bias and concerns regarding applicability for each included study. This tool was piloted independently by the same 2 reviewers on 3 studies of diagnostic test accuracy involving other foot and ankle pathologies to determine agreement.^48^ QUADAS-2 includes 4 domains: patient selection, index tests, reference standards, and flow and timing. Risk of bias is assessed for each domain and rated as either “low,” “high,” or “unclear.” Concerns regarding applicability are assessed for the first 3 domains and are rated in the same way. Studies rated “low” in all domains are considered to have an overall “low risk of bias” or “low concern regarding applicability.” Studies rated “high” or “unclear” in 1 or more domains are considered “at risk of bias” or as having “concerns regarding applicability.”^48^ Disagreements between reviewers were resolved through consensus, with a third reviewer available to make a final decision if necessary.

### Data Analysis

Diagnostic 2 × 2 tables were used to calculate sensitivity, specificity, predictive values, and likelihood ratios (LRs) for each subjective feature and clinical examination test in each study.^2,35,42^ Statistical analysis was conducted using MedCalc software (version 19, 2021).^29^ Positive likelihood ratio (LR+) is the ratio of positive test results in people with a condition to positive test results in people without a condition, whereas a negative likelihood ratio (LR–) is the ratio of negative test results in people with a condition to negative test results in people without a condition.^14^ An LR above 1 indicates that a test result is associated with the presence of a condition, whereas an LR below 1 indicates that the test result is associated with the absence of a condition.^14^ The further LRs are from 1, the stronger the evidence for the presence or absence of a condition.^14^ Power et al^37^ suggest that for a test to be considered “useful,” combined sensitivity + specificity values should be at least 150%.

## Results

### Study Selection

**Figure 1.**
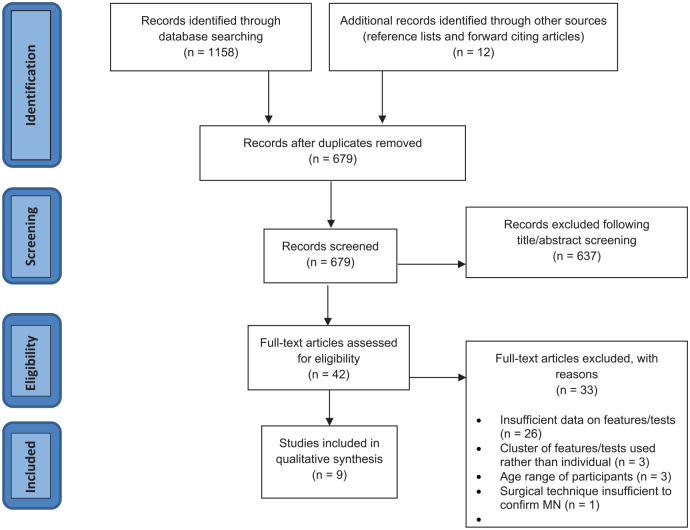
PRISMA flow diagram.

Database searching resulted in a total of 1158 articles, with a further 12 studies found through hand searching. After screening, 42 full texts were retrieved and reviewed, with 9 studies meeting the inclusion criteria (Figure 1).

### Study Quality

Full details of risk of bias and applicability assessments completed using QUADAS-2 can be seen in Table 1. Only 1 study was considered overall to be at low risk of bias,^12^ whereas only 2 studies were considered to have overall low concerns regarding applicability.^12,26^

**Table 1.** QUADAS-2 Results.^a^

	Risk of Bias					Concerns Regarding Applicability			
Study	Patient Selection	Index Test	Reference Standard	Flow and Timing	Overall Risk of Bias	Patient Selection	Index Test	Reference Standard	Overall Applicability concerns
Aydinlar et al^3^	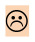	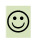	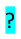	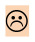	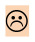	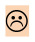	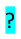	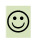	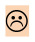
Çelik et al^10^	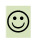	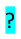	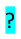	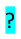	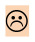	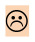	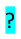	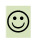	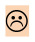
Cloke et al^11^	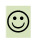	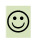	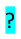	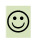	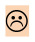	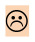	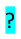	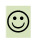	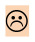
Dando^12^	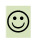	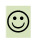	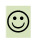	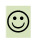	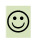	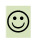	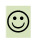	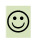	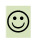
Giannini et al^17^	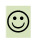	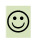	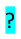	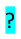	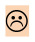	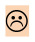	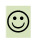	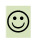	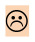
Mahadevan et al^26^	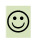	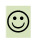	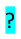	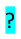	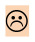	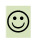	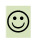	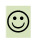	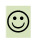
Mann et al^27^	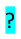	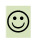	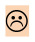	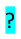	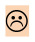	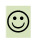	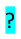	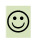	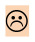
Owens et al^33^	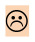	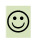	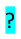	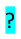	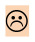	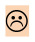	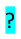	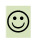	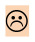
Pastides et al^36^	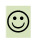	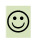	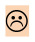	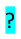	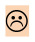	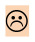	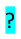	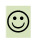	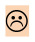

a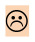, low risk of bias; 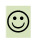, high risk of bias; 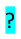, unclear whether low or high risk of bias.

### Study Demographics

Table 2 reports participant and study details for each included study. Aydinlar et al^3^ included 15 participants; however, 3 of these were excluded from the analysis as 1 did not undergo a reference standard whereas 2 had bilateral symptoms and it was not clear which symptoms related to which foot. Mann and Reynolds^27^ included 56 participants with 76 neuromas; however, 11 neuromas were recurrent following a previous operation and were therefore excluded from the current analysis. Owens et al^33^ included a control group (Table 2, group B); however, these patients were not suspected of having MN and were therefore excluded from the analysis.

**Table 2.** Study Demographics.

Study	Participants	Years of Age, Mean (range)	Country of Study	Reference Standard	Symptom Duration, Mean (range)	Exclusion Criteria	Primary Purpose of Study	Assessment Completed By	Data Collection
Aydinlar et al^3^	N = 15 (F 10, M 5)	49 (25-70)	Turkey	MRI	NR	NR	Use of electrodiagnostics for diagnosis of MN	1 neurologist	Prospective cohort study
Çelik et al^10^	N = 27 (F 21, M 6)	49 (24-74)	Turkey	Surgery	NR	Other anatomical forefoot pathology	Patient function following MN surgery	NR	Retrospective notes review
Cloke et al^11^	N = 22 (F 19, M 3)	51 (20-80)	United Kingdom	Surgery	NR	NR	Diagnostic accuracy of physical examination tests for MN	1 surgeon plus an “observer”	Prospective cohort study
Dando^12^	N = 30 (F 18, M 12)	58 (37-81)	United Kingdom	Ultrasonography	3.8 y (2 mo–15 y)	Rearfoot or midfoot pain	Reliability and validity of a clinical assessment protocol for MN	1 podiatrist	Prospective cohort study
Giannini et al^17^	N = 60 (63 neuromas) (F 55, M 5)	49 (20-76)	Italy	Surgery	NR	NR	Clinical results following MN surgery	NR	Retrospective notes review
Mahadevan et al^26^	N = 40 (54 neuromas) (F 31, M 9)	54 (26-74)	United Kingdom	Ultrasonography	2.3 y (2 mo– 10 y)	Peripheral neuropathy, significant hallux valgus and/or deformity of the lesser toes, metatarsophalangeal joint subluxation or instability, previous forefoot surgery, inflammatory arthropathy	Diagnostic accuracy of physical examination tests for MN	1 surgeon	Prospective cohort study
Mann et al^27^	N = 56 (76 neuromas) (F 53, M 3)	55 (29-81)	USA	Surgery	NR (2 months – 15 years)	NR	Reporting of subjective symptoms pre and post MN surgery	NR	Retrospective notes review
Owens et al^33^	Group A N = 70 (76 neuromas) (F 60, M 10) Group B N = 34 (F NR, M NR)	51 (27-73)	United Kingdom	Surgery	NR	Rheumatoid arthritis	Diagnostic accuracy of physical examination tests for MN compared with controls	NR	Retrospective notes review
Pastides et al^36^	N = 36 (43 neuromas) (F 28, M 8)	43.8 (25-71)	United Kingdom	Surgery	NR	NR	Accuracy and correlation of clinical, radiological and operative findings in MN	NR	Retrospective notes review

Abbreviations: F, female; M, male; MN, Morton neuroma; MRI, magnetic resonance imaging; NR, not reported.

Tables 3 and 4 report diagnostic accuracy of subjective features and physical examination tests, respectively. In studies where sensitivity, specificity, PPV, NPV, LR+, and LR– were not reported, these were calculated for this review. Cloke and Greiss considered index tests to be positive if patients had either MN or neuritis.^11^ The authors were contacted, and raw data obtained to ascertain which patients had MN and which had neuritis. Only patients with confirmed MN were considered positive for the purposes of this review, hence the difference in results presented here and those in the original study.

**Table 3.** Diagnostic Accuracy of Subjective Features for MN.

Subjective Feature	Study	TP	FP	FN	TN	Sensitivity (95% CI)	Specificity (95% CI)	PPV (95% CI)	NPV (95% CI)	LR+ (95% CI)	LR– (95% CI)
Description of symptoms											
Burning	Dando^12^	4	12	3	11	0.57 (0.18-0.90)	0.48 (0.27-0.69)	0.25 (0.14-0.41)	0.79 (0.59-0.91)	1.10 (0.52-2.32)	0.90 (0.34-2.33)
	Mann et al^27^	35	0	30	0	0.54 (0.41-0.66)	N/A	1.00 (0.94-1.00)	0.00	N/A	N/A
Clicking	Dando^12^	4	1	3	22	0.57 (0.18-0.90)	0.96 (0.78-1.00)	0.80 (0.35-0.97)	0.88 (0.76-0.95)	13.14 (1.74-99.2)	0.45 (0.19-1.06)
Cramping sensation	Mann et al^27^	22	0	43	0	0.34 (0.23-0.47)	N/A	1.00 (0.85-1.00)	0.00	N/A	N/A
Feeling of a pebble in shoe	Dando^12^	3	11	4	12	0.43 (0.10-0.82)	0.52 (0.31-0.73)	0.21 (0.10-0.42)	0.75 (0.59-0.86)	0.90 (0.34-2.33)	1.10 (0.52-2.32)
	Mahadevan et al^26^	28	0	25	0	0.53 (0.39-0.67)	N/A	1.00 (0.88-1.00)	0.00	N/A	N/A
Shooting pain	Dando^12^	5	14	2	9	0.71 (0.29-0.96)	0.39 (0.20-0.62)	0.26 (0.17-0.39)	0.82 (0.56-0.94)	1.17 (0.66-2.08)	0.73 (0.20-2.62)
Sensation											
Altered sensation (location unspecified)	Mahadevan et al^26^	32	0	21	0	0.60 (0.46-0.74)	N/A	1.00 (0.89-1.00)	0.00	N/A	N/A
Dysesthesia Numbness (location unspecified)	Çelik et al^10^	0	0	27	0	0.00 (0.00-0.13)	N/A	N/A	0.00	N/A	N/A
	Çelik et al^10^	9	0	18	0	0.33 (0.17-0.54)	N/A	1.00 (0.66-1.00)	0.00	N/A	N/A
Numbness in toes or foot Paraesthesia (location unspecified)	Mann et al^27^	26	0	39	0	0.40 (0.28-0.53)	N/A	1.00 (0.87-1.00)	0.00	N/A	N/A
	Aydinlar et al^3^	9	0	3	0	0.75 (0.43-0.95)	N/A	1.00 (0.66-1.00)	0.00	N/A	N/A
Paraesthesia into toes Pins and needles (location unspecified)	Dando^12^	4	12	3	11	0.57 (0.18-0.90)	0.48 (0.27-0.69)	0.25 (0.14-0.41)	0.79 (0.59-0.91)	1.10 (0.52-2.32)	0.90 (0.34-2.33)
	Dando^12^	6	13	1	10	0.86 (0.42-1.00)	0.43 (0.23-0.64)	0.32 (0.22-0.43)	0.91 (0.61-0.99)	1.52 (0.95-2.42)	0.33 (0.05-2.14)
Footwear											
Fashionable shoes agg.	Giannini et al^17^	46	0	17	0	0.73 (0.60-0.83)	N/A	1.00 (0.92-1.00)	0.00	N/A	N/A
Pain agg. by tight shoes	Dando^12^	6	10	1	13	0.86 (0.42-1.00)	0.57 (0.35-0.77)	0.38 (0.26-0.51)	0.93 (0.67-0.99)	1.97 (1.13-3.44)	0.25 (0.04-1.61)
	Pastides et al^36^	37	0	6	0	0.86 (0.72-0.95)	N/A	1.00 (0.91-1.00)	0.00	N/A	N/A
Shoes agg.	Mahadevan et al^26^	40	0	13	0	0.75 (0.62-0.86)	N/A	1.00 (0.91-1.00)	0.00	N/A	N/A
Shoes agg. when walking	Giannini et al^17^	63	0	0	0	1.00 (0.94-1.00)	N/A	1.00 (0.94-1.00)	N/A	N/A	N/A
Location of pain											
Forefoot pain	Dando^12^	7	20	0	3	1.00 (0.59-1.00)	0.13 (0.03-0.34)	0.26 (0.23-0.29)	1.00 (0.29-1.00)	1.15 (0.98-1.35)	N/A
Pain in second/third IM space	Dando^12^	6	12	1	11	0.86 (0.49-0.97)	0.48 (0.29-0.67)	0.33 (0.14-0.59)	0.92 (0.60-1.00)	1.64 (1.00-2.69)	0.30 (0.05-1.93)
Pain radiating to toes	Mann et al^27^	40	0	25	0	0.62 (0.49-0.72)	N/A	1.00 (0.89-1.00)	0.00 (0.00-0.16)	N/A	N/A
Pain up foot or leg	Mann et al^27^	22	0	43	0	0.34 (0.24-0.46)	N/A	1.00 (0.82-1.00)	0.00 (0.00-0.10)	N/A	N/A
Plantar foot pain	Mann et al^27^	50	0	15	0	0.77 (0.65-0.85)	N/A	1.00 (0.91-1.00)	0.00 (0.00-0.25)	N/A	N/A
Relieving factors											
Massage	Mahadevan et al^26^	15	0	38	0	0.28 (0.18-0.42)	N/A	1.00 (0.75-1.00)	0.00 (0.00-0.11)	N/A	N/A
Removing shoes	Mahadevan et al^26^	30	0	23	0	0.57 (0.43-0.69)	N/A	1.00 (0.86-1.00)	0.00 (0.00-0.18)	N/A	N/A
	Mann et al^27^	46	0	19	0	0.71 (0.59-0.80)	N/A	1.00 (0.90-1.00)	0.00 (0.00-0.21)	N/A	N/A
Rest	Mahadevan et al^26^	44	0	9	0	0.83 (0.71-0.91)	N/A	1.00 (0.90-1.00)	0.00 (0.00-0.37)	N/A	N/A
	Mann et al^27^	58	0	7	0	0.89 (0.79-0.95)	N/A	1.00 (0.92-1.00)	0.00 (0.00-0.44)	N/A	N/A
	Pastides et al^36^	35	0	8	0	0.81 (0.67-0.90)	N/A	1.00 (0.88-1.00)	0.00 (0.00-0.40)	N/A	N/A
Walking											
Severe limitation in walking	Çelik et al^10^	21	0	6	0	0.78 (0.59-0.89)	N/A	1.00 (0.81-1.00)	0.00 (0.00-0.48)	N/A	N/A
Pain aggravated by walking	Mahadevan et al^26^	49	0	4	0	0.92 (0.82-0.97)	N/A	1.00 (0.91-1.00)	0.00 (0.00-0.60)	N/A	N/A
	Mann et al^27^	59	0	6	0	0.91 (0.81-0.96)	N/A	1.00 (0.92-1.00)	0.00 (0.00-0.48)	N/A	N/A
	Pastides et al^36^	39	0	4	0	0.91 (0.78-0.96)	N/A	1.00 (0.88-1.00)	0.00 (0.00-0.60)	N/A	N/A

Abbreviations: agg, aggravate; FN, false negative; FP, false positive; IM, intermetatarsal; MN, Morton neuroma; N/A, results not able to be calculated; TN, true negative; TP, true positive.

**Table 4.** Diagnostic Accuracy of Physical Examination Tests for MN.

Physical Examination Test	Study	TP	FP	FN	TN	Sensitivity (95% CI)	Specificity (95% CI)	PPV (95% CI)	NPV (95% CI)	LR+ (95% CI)	LR– (95% CI)
Compression/squeeze tests											
Metatarsal approximation	Cloke et al^11^	14	6	2	0	0.88 (0.64-0.97)	0.00 (0.00-0.39)	0.70 (0.46-0.87)	0.00 (0.00-0.80)	0.88 (0.73-1.05)	N/A
Foot squeeze test	Mahadevan et al^26^	22	1	31	0	0.42 (0.29-0.55)	0.00 (0.00-0.79)	0.96 (0.76-1.00)	0.00 (0.00-0.14)	0.42 (0.30-0.57)	N/A
	Owens et al^33^	66	1	9	0	0.88 (0.79-0.94)	0.00 (0.00-0.79)	0.99 (0.91-1.00)	0.00 (0.00-0.37)	0.88 (0.81-0.96)	N/A
Pain lateral forefoot compression	Dando^12^	1	5	6	18	0.14 (0.03-0.51)	0.78 (0.58-0.90)	0.17 (0.01-0.64)	0.75 (0.53-0.89)	0.66 (0.09-4.73)	1.10 (0.76-1.59)
Pain squeezing individual metatarsal heads	Dando^12^	0	9	7	14	0.00 (0.00-0.35)	0.61 (0.41-0.78)	0.00 (0.00-0.37)	0.67 (0.43-0.85)	0	1.70 (1.18-1.28)
Diastasis between toes	Dando^12^	1	4	6	19	0.14 (0.03-0.51)	0.83 (0.63-0.93)	0.20 (0.01-0.70)	0.76 (0.54-0.90)	0.82 (0.11-6.20)	1.04 (0.73-1.48)
	Mann et al^27^	2	0	63	0	0.03 (0.01-0.11)	N/A	1.00 (0.20-1.00)	0.00 (0.00-0.07)	N/A	N/A
Digital nerve stretch test	Cloke et al^11^	16	6	0	0	1.00 (0.81-1.00)	0.00 (0.00-0.39)	0.73 (0.52-0.87)	N/A	1.00 (1.00-1.00)	N/A
Mass palpated	Mann et al^27^	8	0	57	0	0.12 (0.06-0.22)	N/A	1.00 (0.60-1.00)	0.00 (0.00-0.08)	N/A	N/A
Positive Mulder’s sign	Cloke et al^11^	15	5	1	1	0.94 (0.72-0.99)	0.17 (0.03-0.56)	0.75 (0.51-0.90)	0.50 (0.03-0.97)	1.13 (0.77-1.64)	0.38 (0.03-5.09)
	Dando^12^	2	3	5	20	0.29 (0.08-0.64)	0.87 (0.68-0.95)	0.40 (0.07-0.83)	0.80 (0.59-0.92)	2.19 (0.45-10.60)	0.82 (0.50-1.35)
	Mahadevan et al^26^	34	0	19	1	0.64 (0.51-0.76)	1.00 (0.21-1.00)	1.00 (0.87-1.00)	0.05 (0.00-0.27)	N/A	0.36 (0.25-0.51)
No pain joint margin palp.	Dando^12^	1	5	6	18	0.14 (0.03-0.51)	0.78 (0.58-0.90)	0.17 (0.01-0.64)	0.75 (0.53-0.89)	0.66 (0.09-4.73)	1.10 (0.76-1.59)
No pain joint movement	Dando^12^	1	5	6	18	0.14 (0.03-0.51)	0.78 (0.58-0.90)	0.17 (0.01-0.64)	0.75 (0.53-0.89)	0.66 (0.09-4.73)	1.10 (0.76-1.59)
No swelling	Dando^12^	1	2	6	21	0.14 (0.03-0.51)	0.91 (0.73-0.98)	0.33 (0.02-0.87)	0.78 (0.57-0.91)	1.64 (0.17-15.53)	0.94 (0.68-1.30)
Percussion tests											
Dorsal percussion	Mahadevan et al^26^	17	0	36	1	0.32 (0.21-0.45)	1.00 (0.21-1.00)	1.00 (0.77-1.00)	0.03 (0.00-0.16)	N/A	0.68 (0.57-0.82)
Plantar percussion	Mahadevan et al^26^	19	0	34	1	0.36 (0.24-0.49)	1.00 (0.21-1.00)	1.00 (0.79-1.00)	0.03 (0.00-0.17)	N/A	0.64 (0.53-0.79)
	Owens et al^33^	46	1	29	0	0.61 (0.50-0.72)	0.00 (0.00-0.79)	0.98 (0.87-1.00)	0.00 (0.00-0.15)	0.61 (0.51-0.73)	N/A
Plantar tenderness	Mann et al^27^	62	0	3	0	0.95 (0.87-0.98)	N/A	1.00 (0.93-1.00)	0.00 (0.00-0.69)	N/A	N/A
Sensation tests											
Hypoesthesia with light touch	Aydinlar et al^3^	6	0	6	0	0.50 (0.25-0.75)	N/A	1.00 (0.52-1.00)	0.00 (0.00-0.48)	N/A	N/A
Light touch sensation deficit	Mahadevan et al^26^	13	0	40	1	0.25 (0.15-0.38)	1.00 (0.21-1.00)	1.00 (0.72-1.00)	0.02 (0.00-0.14)	N/A	0.76 (0.65-0.88)
Pin prick sensation deficit	Mahadevan et al^26^	13	0	40	1	0.25 (0.15-0.38)	1.00 (0.21-1.00)	1.00 (0.72-1.00)	0.02 (0.00-0.14)	N/A	0.76 (0.65-0.88)
Toe tip sensation deficit	Owens et al^33^	37	0	38	1	0.49 (0.38-0.60)	1.00 (0.21-1.00)	1.00 (0.88-1.00)	0.03 (0.00-0.15)	N/A	0.51 (0.41-0.63)
Webspace tenderness tests											
Webspace tenderness	Cloke et al^11^	15	6	1	0	0.94 (0.72-0.99)	0.00 (0.00-0.39)	0.71 (0.48-0.88)	0.00 (0.00-0.95)	0.94 (0.83-1.06)	N/A
	Dando^12^	4	16	3	7	0.57 (0.25-0.84)	0.30 (0.16-0.51)	0.20 (0.07-0.44)	0.70 (0.35-0.92)	0.82 (0.41-0.65)	1.41 (0.49-4.05)
Modified webspace tenderness test (thumb index finger squeeze test)	Giannini et al^17^	43	0	20	0	0.68 (0.56-0.78)	N/A	1.00 (0.90-1.00)	0.00 (0.00-0.20)	N/A	N/A
	Owens et al^33^	71	1	4	0	0.95 (0.87-0.98)	0.00 (0.00-0.79)	0.99 (0.91-1.00)	0.00 (0.00-0.60)	0.95 (0.87-0.98)	N/A
	Pastides et al^36^	43	0	0	0	1.00 (0.91-1.00)	N/A	1.00 (0.90-1.00)	N/A	N/A	N/A
	Mahadevan et al^26^	51	0	2	1	0.96 (0.87-0.99)	1.00 (0.21-1.00)	1.00 (0.91-1.00)	0.33 (0.02-0.87)	N/A	0.04 (0.01-0.15)

Abbreviations: FN, false negative; FP, false positive; MN, Morton neuroma; N/A, results not able to be calculated; palp., palpation; TN, true negative; TP, true positive.

Twenty-six different subjective features (Table 3) and 20 different physical examination tests (Table 4) were reported. *Pain aggravated by walking* and *pain relieved by rest* were the most commonly included subjective features (3 studies) whereas the most commonly included physical examination test was webspace tenderness (5 studies), as well as a modified version (thumb index finger squeeze test) devised by Mahadevan et al.^26^

### Sensitivity and Specificity

Sensitivity of subjective features ranged from 0% for dysesthesias^10^ to 100% for forefoot pain^12^ and shoes aggravating while walking.^17^ Other studies also reported high sensitivity (91%-92%) for walking aggravating pain^26,27,36^ as well as 75%-86% for footwear aggravating pain.^12,17,26,36^ Specificity of subjective features ranged from 13% for forefoot pain to 96% for clicking.^12^

Sensitivity of physical examination tests ranged from 0% for pain on squeezing individual metatarsal heads^12^ to 100% for the digital nerve stretch test^11^ and webspace tenderness.^36^ Webspace tenderness sensitivity ranged from 57% to 95% across 4 other studies.^11,12,17,33^ Several physical examination tests had a specificity of 0% or 100% because of 7 studies having only 1 or zero participants without MN (Table 4).

### Likelihood Ratios

Clicking reported by the patient was the only subjective feature with an LR+ greater than 10 (LR 13.14),^12^ which is considered strong evidence for presence of MN. No other subjective feature LR+ was greater than 2 and therefore all were considered very weak evidence for presence of MN. Four subjective features (pain located in second or third IM space, pins and needles, clicking, and pain aggravated by tight shoes) had LR– between 0.2 and 0.5,^12^ indicating weak evidence for absence of MN if these symptoms were not present. LR– for all other subjective features were considered very weak evidence for absence of MN.

Mulder’s sign in the study by Dando^12^ was the only physical examination test with an LR+ above 2 (2.19), indicating weak evidence for presence of MN. The modified webspace tenderness test (thumb index finger squeeze test)^26^ had an LR– of 0.04, which is considered strong evidence to rule out MN with a negative test result. The LR– for Mulder’s sign in 2 studies^11,26^ was considered weak evidence; however, it was very weak in a third study.^12^

## Discussion

The aim of this systematic review was to examine the diagnostic accuracy of subjective features and physical examination tests for MN. Because of low numbers of participants without the target condition (ie, MN), specificity and LRs could not be accurately calculated in several studies (Tables 3 and 4). This is an issue reported with similar systematic reviews of diagnostic test accuracy in the hip and foot.^9,40^

### Study Quality

One study had low risk of bias^12^ and 2 had low concerns regarding applicability to clinical practice.^12,26^ These were 2 of 3 prospective cohort studies in which diagnostic accuracy of features and tests for MN was the primary objective. Applicability of the index tests was often unclear in surgical studies because of the lack of clarity regarding the time between the index test and the reference standard. This is especially important as once surgical intervention is considered, a patient is likely to have *failed* conservative management. As a result, assessment may not be taking place at the same time in the course of the condition as it would in clinical practice, and therefore prospective studies using noninvasive examination techniques may be more representative of clinical practice.

### Subjective Features

Subjective clicking reported by the patient was highly specific (96%) with an LR+ above 10 (13.14)^12^ indicating strong evidence to rule in MN with a positive result. Participants in the study were asked whether they experienced any clicking in the forefoot. Clicking was only included in 1 study; however, the result is more robust as the study had both low risk of bias and low concerns regarding applicability.^12^

Both a feeling of *walking on a pebble* and *burning pain* are regularly mentioned in the literature as common features of MN.^5,34,46^ Sensitivity for these features was 43% to 53% and 54% to 57%, respectively, with associated specificity of 52% and 48%.^12,26,27^ Combined sensitivity + specificity failed to reach 150% for both features, whereas LR+ and LR– were both considered very weak evidence to rule MN either in or out.^12^ This indicates that neither of these subjective features may be considered useful for diagnosis. Two of the 3 studies that included these features were prospective cohort studies with low concerns regarding applicability^12,26^ and appear to highlight a difference between research and clinical practice.

Pain relieved by rest and pain aggravated by walking were both included in 3 studies.^26,27,36^ Sensitivity ranged from 81% to 89% for rest relieving pain and from 91% to 92% for pain aggravated by walking. Unfortunately, specificity values and LRs could not be accurately calculated for any of these studies because of low numbers of participants without MN in each of these studies.

During subjective assessment, clicking reported by the patient appears to be clinically useful in the diagnosis of MN, whereas a feeling of walking on a pebble or a burning pain in the foot had lower diagnostic accuracy.

### Physical Examination Tests

Positive Mulder’s sign, foot squeeze test, and webspace tenderness test are considered key physical examination tests for assessment of MN by the American College of Foot and Ankle Surgeons,^45^ the Association of Extremity Nerve Surgeons,^4^ and a Delphi study consensus statement.^13^ Results from the current systematic review suggest sensitivity of Mulder’s sign varied from 29% to 94% and specificity from 17% to 100%.^11,12,26^ Specificity values were highest (87%-100%) in the latter 2 studies, which have lower risk of bias^12,26^ ; however both LR+ and LR– were considered weak or very weak evidence to rule MN in or out. LRs are considered one of the best indicators of diagnostic accuracy of clinical tests^21,42^ and take into account both sensitivity and specificity values and therefore have a higher relevance to clinical practice than other statistics.^14^ Therefore, although some results appear to support the use of Mulder’s sign, those with higher relevance to clinical practice do not. This finding supports previous research with Mulder sign found to be positive in as low as 40% of patients with MN in other studies.^33^

Sensitivity of foot squeeze tests varied from 0% to 88% and specificity from 0% to 78%, with no study reporting a sensitivity + specificity value indicating clinical usefulness, and all LR+ and LR– considered very weak evidence to rule MN in or out.^12,26,33^ Despite being widely used within the literature and recommended by a panel of experts^13^ the results of this systematic review do not support the use of this test to rule MN in or out.

Three studies reported sensitivity values of 94% or above for webspace tenderness; however, these studies were all considered at risk of bias as well as having concerns regarding applicability.^11,33,36^ LR+ and LR– were considered very weak evidence to rule MN either in or out in all studies.^11,12,17,33,36^ Mahadevan et al^26^ reported 100% specificity with the use of a modified version of the webspace tenderness test (thumb index finger squeeze test) and LR– (0.04) was considered strong evidence to rule out MN with a negative test. Modification involved the use of the thumb pad^26^ rather than the side of the thumb as described in other articles,^17,33^ whereas performance differs from Mulder’s sign as no lateral compression is applied to the foot. Some might consider the performance of the modified version not that dissimilar to the webspace tenderness test; however, given the differing results, further studies directly comparing the two are warranted.

### Strengths and Limitations

Attempts were made to reduce language bias, and thorough hand-searching of reference lists and forward citing articles was completed to ensure all appropriate studies were included. Subjective features are an important aspect of diagnosis^5,15,18^ and their inclusion in this systematic review increases the relevance to clinical practice.

A limitation is the number of studies that included few or no patients without MN, and the impact this had on the ability to calculate specificity values and LRs, or confidently interpret predictive values. This was a result of the number of studies that were retrospective surgical studies rather than prospective diagnostic criterion validity studies. Additionally, the QUADAS-2 tool has not been widely used to assess subjective features in the literature and therefore further reliability and validity testing of QUADAS-2 for subjective features may be appropriate in the future.

## Conclusions

The diagnostic accuracy of subjective features and physical examination tests for MN is variable. There is strong evidence that *clicking* reported by a patient rules in MN and that the modified webspace tenderness test, when negative, rules out MN. It should be noted, however, that both of these results come from single studies. Timely accurate clinical diagnosis is particularly important given the cost implications associated with imaging modalities and financial pressure on the National Health Service. The results of this systematic review raise questions about the diagnostic accuracy of subjective features such as burning pain and a feeling of walking on a pebble, as well as physical examination tests such as Mulder’s sign, foot squeeze tests, and webspace tenderness that are commonly cited within the MN literature. Caution should be exercised because of methodological limitations of some studies, and prospective studies with larger populations including patients both with and without MN are required to direct clinicians with more conviction.

## Supplemental Material

sj-pdf-1-fao-10.1177_24730114241291055 – Supplemental material for Diagnostic Accuracy of Subjective Features and Physical Examination Tests for Morton Neuroma: A Systematic Review Supplemental material, sj-pdf-1-fao-10.1177_24730114241291055 for Diagnostic Accuracy of Subjective Features and Physical Examination Tests for Morton Neuroma: A Systematic Review by Mark Pitcher, Andrea Moulson, David Pitcher, Anthony Herbland, Grad Cert and Mindy C. Cairns in Foot & Ankle Orthopaedics

## Appendix A

**Table:**

Search strategy for PsycINFO database	
1	morton’s neur*.mp. [mp = title, abstract, heading word, table of contents, key concepts, original title, tests, measures, mesh]
2	interdigital neur*.mp.
3	intermetatarsal neur*.mp.
4	forefoot neur*.mp.
5	morton’s metat*.mp.
6	plantar digital neur*.mp.
7	plantar interdigital neur*.mp.
8	morton’s entrapment.mp.
9	interdigital nerve*.mp.
10	1 OR 2 OR 3 OR 4 OR 5 OR 6 OR 7 OR 8 OR 9
11	test*.mp.
12	exam*.mp.
13	symptom*.mp.
14	present*.mp.
15	sign*.mp.
16	eval*.mp.
17	feature*.mp.
18	11 OR 12 OR 13 OR 14 OR 15 OR 16 OR 17
19	imag*.mp.
20	surg*.mp.
21	ultras*.mp.
22	MRI*.mp.
23	magnetic resonance imagin*.mp.
24	arthrosco*.mp.
25	anaesth*.mp.
26	inject*.mp.
27	ster*.mp.
28	corticoster*.mp.
29	19 OR 20 OR 21 OR 22 OR 23 OR 24 OR 25 OR 26 OR 27 OR 28
30	valid*.mp.
31	accura*.mp.
32	sensitiv*.mp.
33	specific*.mp.
34	reliab*.mp.
35	diagn*.mp.
36	30 OR 31 OR 32 OR 33 OR 34 OR 35
37	10 AND 18 AND 29 AND 36
